# Understanding Scanner Utilization With Real-Time DICOM Metadata Extraction

**DOI:** 10.1109/access.2021.3050467

**Published:** 2021-01-11

**Authors:** PRADEEBAN KATHIRAVELU, ASHISH SHARMA, PUNEET SHARMA

**Affiliations:** 1 Department of Biomedical Informatics, Emory University, Atlanta, GA 30322, USA; 2 Department of Radiology and Imaging Sciences, Emory University, Atlanta, GA 30322, USA

**Keywords:** Biomedical imaging, digital imaging and communications in medicine (DICOM), metadata extraction, picture archiving and communication system (PACS), scanner utilization

## Abstract

Understanding system performance metrics ensures better utilization of the radiology resources with more targeted interventions. The images produced by radiology scanners typically follow the DICOM (Digital Imaging and Communications in Medicine) standard format. The DICOM images consist of textual metadata that can be used to calculate key timing parameters, such as the exact study durations and scanner utilization. However, hospital networks lack the resources and capabilities to extract the metadata from the images quickly and automatically compute the scanner utilization properties. Thus, they resort to using data records from the Radiology Information Systems (RIS). However, data acquired from RIS are prone to human errors, rendering many derived key performance metrics inadequate and inaccurate. Hence, there is motivation to establish a real-time image transfer from the Picture Archiving and Communication Systems (PACS) to receive the DICOM images from the scanners to research clusters to conduct such metadata processing to evaluate scanner utilization metrics efficiently and quickly. This paper analyzes the scanners’ utilization by developing a real-time monitoring framework that retrieves radiology images into a research cluster using the DICOM networking protocol and then extracts and processes the metadata from the images. Our proposed approach facilitates a better understanding of scanner utilization across a vast healthcare network by observing properties such as study duration, the interval between the encounters, and the series count of studies. Benchmarks against using the RIS data indicate that our proposed framework based on real-time PACS data estimates the scanner utilization more accurately. Furthermore, our framework has been running stable and performing its computation for more than two years on our extensive healthcare network in pseudo real-time.

## I. INTRODUCTION

Patient exam times and scanner utilization are key indicators of efficiency and quality in an imaging department [1]. Stake-holders rely on various data sources but mostly depend on the reports obtained from the Radiology Information Systems (RIS) [2]. However, RIS data related to system activity are entered by humans and are prone to errors. Alternatively, research has looked into using the Picture Archiving and Communication Systems (PACS) [3] data to understand scanner utilization. Although promising, such research is limited to the information readily available from the DICOM (Digital Imaging and Communications in Medicine) [4] headers and do not account for specific scenarios such as multi-exam encounters. Real-time extraction and further processing of DICOM headers may provide the best of both worlds, with rich information from the DICOM headers combined with additional computations at a research cluster to understand the scanner utilization properties. With precise knowledge of imaging events and utilization over time across a healthcare network, appropriate intervention can be implemented and monitored consistently.

### A. DIRECTLY ACCESS EACH SCANNER

Understanding each scanner’s performance metrics by directly analyzing the scanners can be the fastest option to compute scanner utilization as we can perform these computations at the acquisition time of an image. However, direct access and processing from the scanners are more challenging in practice as scanners are not optimized for such computations. Understanding the scanner as a stand-alone entity does not provide adequate measures to quantitatively and comparatively understand the scanner utilization across the site and the healthcare network. Consequently, the processed data will still need to be sent to a centralized location to compare all the scanners of the same modality. Using log files from the scanners have been proposed to estimate and improve their utilization [5]. However, accessing the scanner logs is not scalable for an extensive healthcare network due to the large volume of the scanners and the effort required to configure them, especially given the diverse access interfaces, vendor incompatibility [6], and various log formats dependent on modalities and vendors.

### B. RECORDS FROM RIS

Currently, healthcare environments widely use records from RIS to identify and understand the scanner utilization. Most RIS provides accurate high-level exam details, such as patient, scheduling, study, and modality details, but supply inexact data acquisition timestamps due to manually logging by imaging personnel [2]. While extensive, the RIS reports are usually prone to human errors in recording timestamps, particularly the start and end times of a study. Furthermore, RIS does not consist of elaborative image-specific information that is readily available in the DICOM headers. Therefore, the scope and potential of using RIS to identify the scanners’ operational and performance metrics are limited.

### C. DICOM METADATA HEADERS FROM THE PACS

An alternate datasource for timestamp analytics is to use the DICOM imaging data received from all the scanners into a PACS [7]. To implement an effective change like optimized time slots, one must accurately track exam durations for specific study types across multiple sites. The task of monitoring exam durations across numerous devices spanning several sites requires the specificity of exam details, such as exam start and end times, among others. Alternatively, PACS offers a more reliable source for imaging activity occurring at the modality itself. PACS can receive, store, and transfer images from scanners of various modalities in real-time [8]. The medical images themselves, stored with the DICOM format, contain textual metadata, detailing standardized acquisition information [9].

Studies based on DICOM headers provide better understanding and computational accuracy to scanner utilization metrics and patient experience than those using RIS [10]. The richness of DICOM headers has motivated developers to mine DICOM metadata for quantitative measures of exam duration and other timing metrics [11]. With the benefits of this added specificity, several vendors have also begun adopting DICOM metadata analysis into commercial products [12]. Several properties, such as exam duration, exam interval, and system utilization, can be readily calculated by developing scalable algorithms to categorize the array of DICOM metadata fields. But it is essential that such automated processes closely capture the actual imaging scenario at the modality, such as unexpected time delays and multi-exam encounters.

### D. OBJECTIVE

The primary objective of this study is to develop automated and pseudo real-time DICOM metadata extraction and scanner utilization computation algorithms to measure exam durations, exam intervals, and utilization for scanners across a large academic enterprise. Our novel framework processes PACS data and accounts for multi-exam scenarios and intra-study delays. For comparison, we correlate our framework’s estimations of exam volume, patient encounters, and exam timing events with the equivalent data sourced from RIS. Although our framework collects DICOM images and extracts metadata from scanners of various modalities, we limit our focus to MR scanners in the evaluations.

### E. MAJOR CONTRIBUTIONS

In the next sections, we elaborate on our significant contributions in detail. First, we define the metrics to understand scanner utilization. Second, we build a framework to transfer the DICOM imaging data and extract and process the metadata from the images. Third, we propose efficient algorithms to compute the scanner utilization metrics in pseudo real-time. We implement *Niffler*,^1^ an open-source framework that retrieves DICOM images in real-time from the PACS and extracts and processes the metadata to identify several system performance metrics. The architecture of the *Niffler* core framework has been presented in detail in our pre-print [13]. This paper focuses specifically on using DICOM metadata obtained continuously in real-time from the PACS with *Niffler* to understand scanner utilization metrics.

The rest of the paper elaborates on how we can understand scanner utilization and optimize the performance metrics with real-time DICOM metadata extraction. Section II presents the background and state-of-the-art. Section III presents our approach and the architecture that makes it possible to process the metadata in real-time from research clusters on scanner utilization metrics. Section IV evaluates the proposed approach and benchmarks against the observations from RIS. Section V discusses and analyzes the findings in detail, including the limitations of the proposed approach. Finally, Section VI concludes the paper with a summary of the research and future work.

## II. BACKGROUND

Patient imaging access and exam duration significantly impact patient satisfaction and the effectiveness of care [14]. Research studies have looked into reducing the individual scan duration and the overall exam duration [15]. Understanding scanner utilization metrics enables the efficient scheduling of patients and provides better care with minimal wait times [16] while enhancing operational efficiency, such as minimizing the power usage of the scanners [17]. Accurate exam details must be collected and analyzed to address care quality, radiologists’ workload, system utilization, and patient experience [18]. In addition to exam volume and study characteristics, quantitative data such as imaging timestamps are essential to characterize daily imaging events encountered by patients [10].

Efficient scanner utilization depends on several factors. First, optimal scheduling of the patients to a scanner of the respective modality is crucial. The scanners may be distributed across several sites of the same healthcare network. Based on the available resources, the scans can be scheduled accordingly to minimize the wait times. Second, the intervals between scans should be minimized to ensure that the scanners can scan several studies per day as needed. The intervals include those between pairs of series in a study and between the scanner’s consecutive studies. In addition to the intervals, metrics such as the number of studies, encounters, and patients seen by a scanner in a given day or a timeframe, as well as the scan duration per study and overall scanner utilization, are important metrics that identify the performance of a scanner.

The encounters consist of a single study or multiple consecutive studies from the same patient scanned by the same scanner with a short interval. The current PACS-based solutions that process DICOM metadata fall short in identifying multi-exam encounters. Correctly grouping the studies to determine the multi-exam encounters is an additional step beyond what can be derived directly from the DICOM headers. As such, the accuracy in understanding the performance and utilization of the scanners entirely with existing PACS-based approaches is questionable and can be as incomplete or inaccurate as using RIS-based data for such metrics.

Research has proposed various optimizations to store and leverage DICOM metadata more efficiently. The multi-series DICOM (MSD) format eliminates duplicates by separating the textual metadata from the binary DICOM data [19]. As DICOM metadata is freely structured, previous works propose to use NoSQL [20] databases such as CouchDB [21] to store it efficiently without imposing limitations on structures that are not inherent to the DICOM metadata format [22]. Furthermore, distributed systems architectures have been proposed to make metadata processing more efficient than the classic centralized execution [23]. While these works aim at providing a better approach to process and store DICOM metadata, they do not offer a unified framework or algorithms to address the shortcomings in using DICOM headers entirely for the scanner utilization computations.

Efficiency metrics consist of several parameters, such as the study duration, scanner utilization, study interval, and series interval [24]. These metrics aim to improve imaging device productivity. For example, previous research optimizes MRI scanner utilization by computing an optimal time slot per scans [25]. However, this work has used patient duration in place of the encounter duration. Such an approach ignores the potential for multiple encounters for the same patient. Moreover, the merging of study descriptions and counts for the multi-study encounters are not presented. This research gap highlights the need for efficient PACS-based scanner utilization computations that also account for nuances such as multi-exam encounters.

Clinical systems, such as RIS and PACS, do not support complex computations on clinical data or the images. Existing works that exploit PACS and RIS data have their limitations, falling short on providing accurate information to understand scanner utilization. We aim for a new perspective into modality activity via real-time capture and analysis of the DICOM image parameters and timestamp information. We thus propose the real-time transfer of DICOM images and a subsequent pseudo real-time extraction of metadata to support user-defined computations, including machine learning algorithms and workflows.

## III. SCANNER UTILIZATION COMPUTATION

In this section, we present our approach to understanding scanner utilization metrics better. We first briefly introduce the *Niffler* architecture, elaborate its scanner utilization work-flow, and then describe its algorithms.

### A. DICOM METADATA EXTRACTION

**FIGURE 1.**
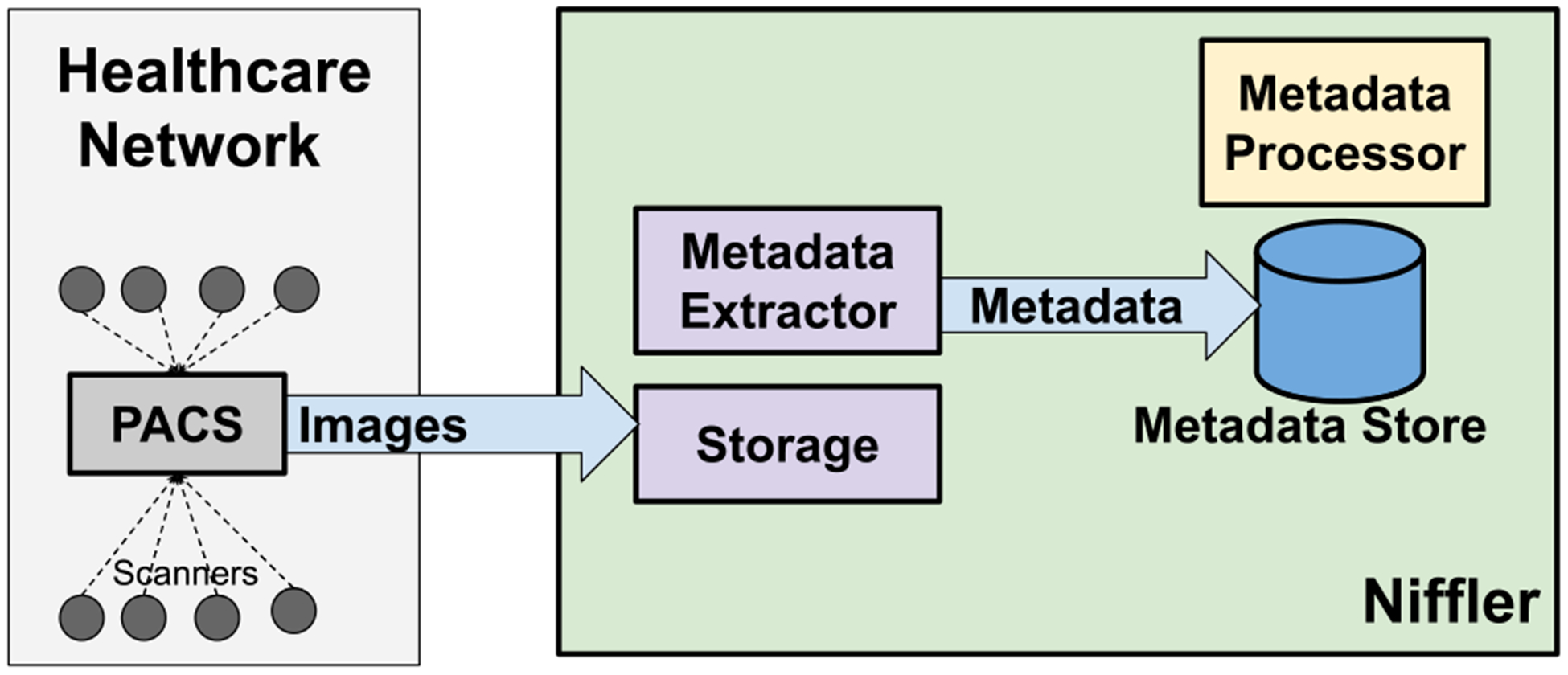
Deployment Architecture.

The scanner utilization computations transform the DICOM metadata attributes to identify the performance metrics. Figure 1 shows the deployment architecture of *Niffler*. Our extensive healthcare network consists of PACS that receive images from the scanners as soon as an exam on a patient produces an image. *Niffler* receives those radiology images from the PACS in real-time as a DICOM stream and stores them temporarily in storage (by default, local file system).

*Niffler* consists of a *Metadata Extractor* that continuously extracts a user-defined subset of metadata attributes from the DICOM images and stores the metadata in pseudo real-time in *Metadata Store*, a scalable and indexed database. *Niffler* uses MongoDB [26], a NoSQL database [20], as its Metadata Store. The metadata attributes to extract are provided by the user as a configuration file consisting of a DICOM header list. *Niffler* periodically deletes those images whose metadata that it has already extracted. A *Metadata Processor* executes on the metadata stored in the Metadata Store to perform user-defined workflows. The scanner utilization computation is such a workflow that runs on the Metadata Store. Given below is a sample entry in the Metadata Store, the metadata attributes extracted from a DICOM image’s headers. Some of the attributes are anonymized for the representation here, stripping off the PHI (Protected health information).


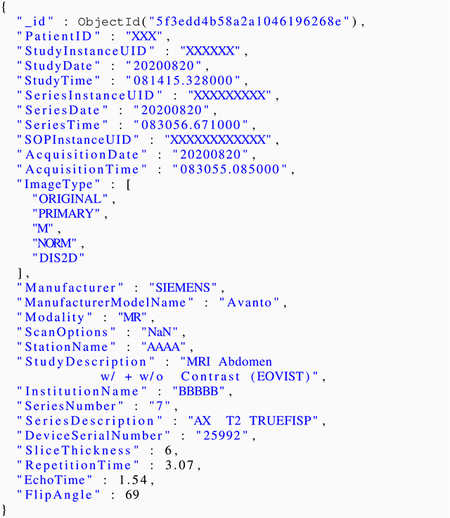


**FIGURE 2.**
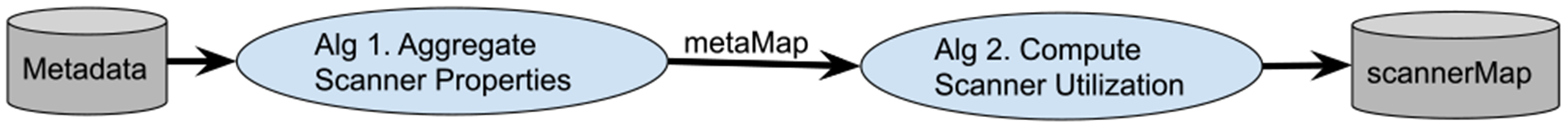
Transformation of the Metadata.

The scanner utilization computation procedure consists of two steps, as Figure 2 illustrates. The first step is a script that transforms the real-time DICOM metadata stored in the metadata store to produce a *metaMap*. The second step is an algorithm that further transforms the processed metadata from the metaMap into a *scannerMap* consisting of scanner utilization properties. We can configure these to run in pseudo real-time: every few minutes to a few hours or nightly to execute on the previous day’s data. Similarly, we can also understand the historical scanner usage patterns over the past several months by analyzing the DICOM metadata belonging to the images acquired in the past, computing the scanner utilization for each day individually for the desired timeframe going backward in the past.

**FIGURE 3.**
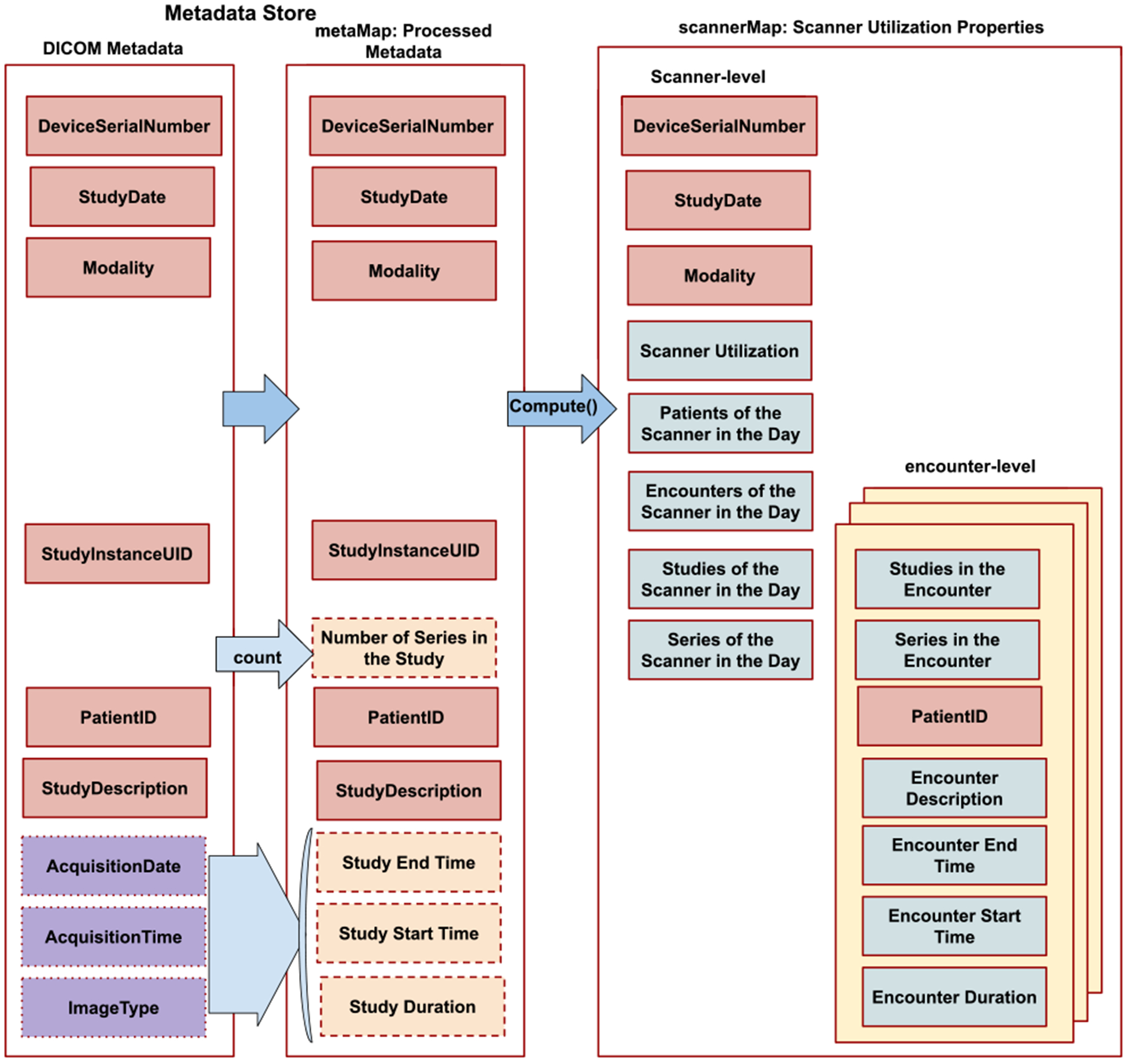
The Metadata Store and the scannerMap.

Figure 3 elaborates properties of the DICOM metadata, metaMap, and scannerMap. The scannerMap consists of a few attributes from the DICOM headers and attributes derived by performing computations on the DICOM metadata attributes. The attributes, *DeviceSerialNumber*, *StudyDate*, and *Modality* are obtained from the metadata and passed on to the scannerMap. *Niffler* calculates the number of series in the study with a simple count of the study’s unique entries of series identifiers.

In its scanner utilization computations, the script considers only the images directly obtained by the scanner and ignores derived images constructed later from the acquired images. Hence, it filters the images by the ImageType attribute, considering only those with the value ‘Primary.’ Eliminating the derived images from the scanner utilization computations is essential as derived images are constructed later and not produced by the scans. *Niffler* counts the number of entries, each represented by a SeriesInstanceUID, to find the number of series in each study. *Niffler* transforms the metaMap to produce the scannerMap consisting of: scanner utilization, as well as the count of patients, encounters, studies, and series belonging to the scanner for the considered duration.

**FIGURE 4.**
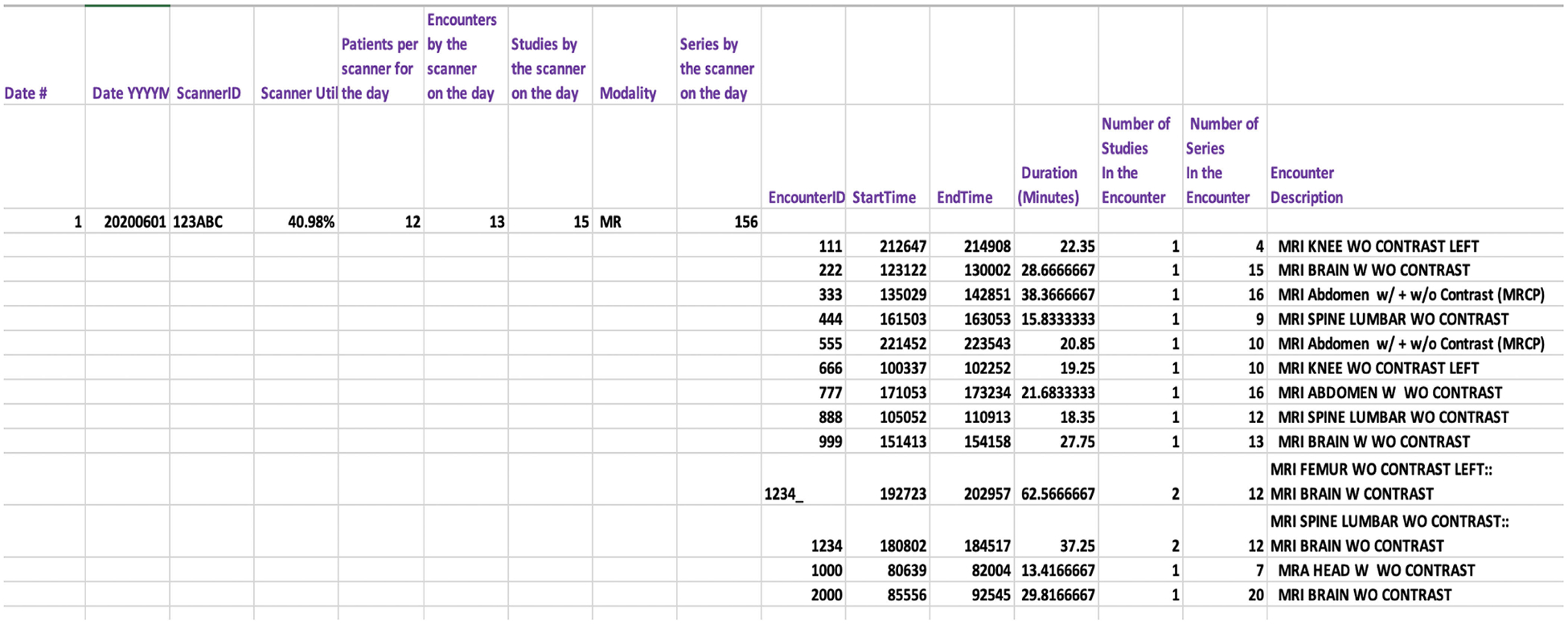
Sample output with the encounter ID and scanner ID anonymized.

In addition to those scanner-level attributes, the scannerMap also consists of several encounter-level attributes such as encounter ID, number of studies and series in the encounter, encounter start and end time, encounter duration, and encounter description. Multi-exam encounters are those which consist of two or more studies. In such encounters, *Niffler* sets the encounter description by merging all the concerning studies’ descriptions. Similarly, *Niffler* computes the number of studies and series in those encounters by adding the number of studies and series in each of the studies that make up the encounter. A *Niffler* algorithm computes the start and end times of the encounter and the scanner utilization metrics by processing the metaMap properties. When a patient has only one encounter, the encounter ID is equal to that of the PatientID. When multiple encounters are present for a single patient, one or more of a special character (by default, ‘_’) is appended to the PatientID to differentiate the subsequent encounter IDs from each other. This naming approach ensures each encounter is uniquely identifiable with capabilities to map and trace back to the respective patient. The scannerMap produces the output grouped for the defined duration, with scanners at the second level. Figure 4 illustrates a segment from the output, showing the data from a scanner for a given day.

By default, *Niffler* computes the properties – the scanner utilization, patients of the scanner, encounters of the scanner, studies of the scanner, and series of the scanner, with a day as the default duration. The scanner utilization workflow runs at 02:00 a.m. every day to calculate the scanner utilization metrics for the day before. *Niffler* uses the study date to consider only the images belonging to the previous date, rather than the acquisition date. Thus, *Niffler* makes sure to consider the studies started on the day before but proceeded to last a few minutes past midnight into the next day as part of the previous day – the day the study began. However, we can configure *Niffler* to calculate the scanner utilization parameters for custom durations at various frequencies.

### B. APPROACH

Each patient visit for a scan by a scanner produces an encounter. The technicians record some encounters as two or more studies when they perform the scan. PACS and other DICOM-based state-of-the-art cannot classify these as a single encounter on their own as DICOM headers alone cannot help group multi-exam encounters. We define ψ as a set composed of all the multi-study encounters (E) and studies (S), as shown by Equation 1.

(1) Ψ={E,S}

**FIGURE 5.**
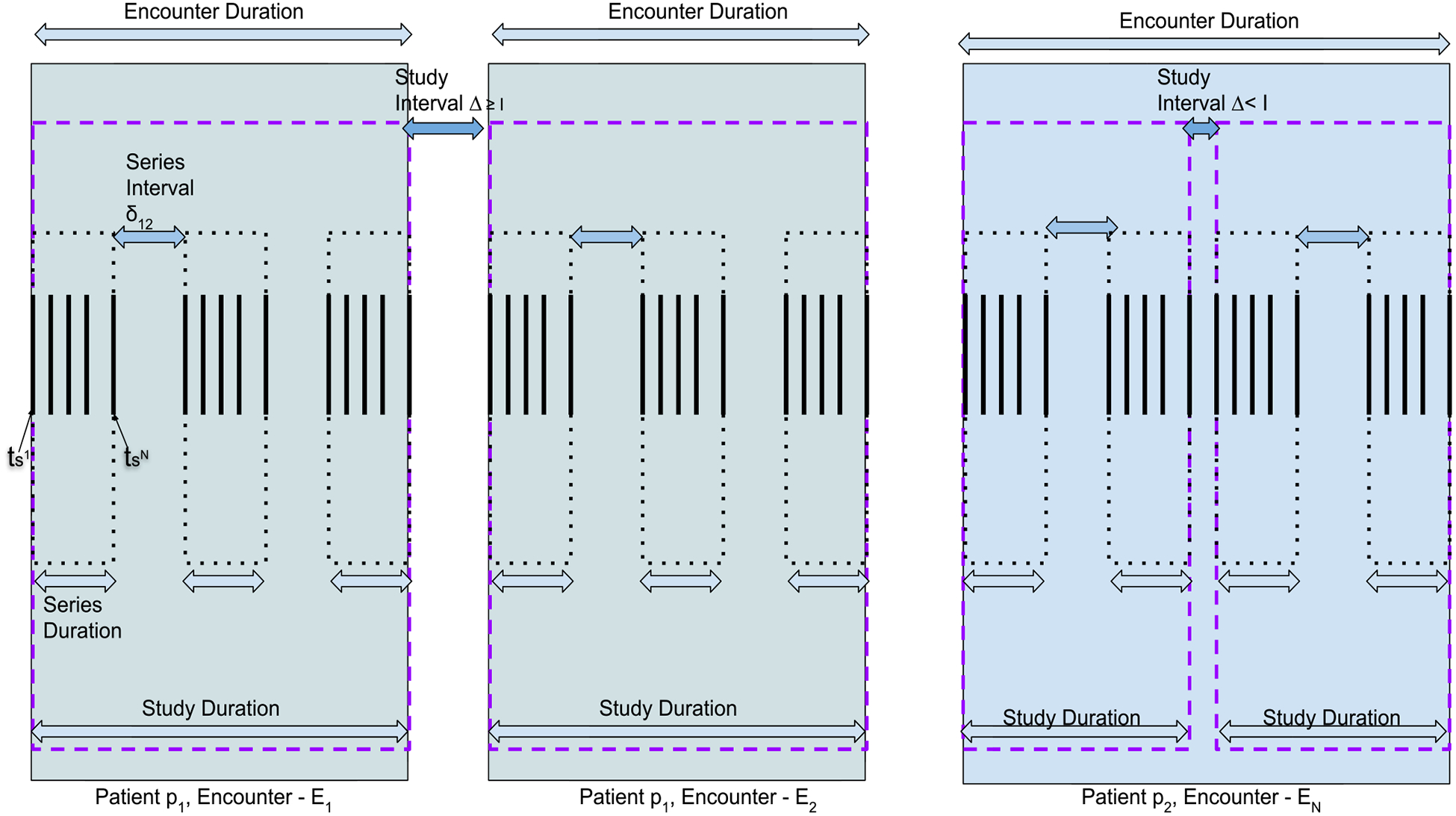
Patients, encounters, and studies by a single scanner.

A scanner performs each study with several series, with several DICOM images acquired for each series. Each DICOM image consists of metadata that provides identifying properties of the images. Figure 5 illustrates the time durations and intervals between the scans. Δ indicates the interval between two studies of the same patient in a given scanner, whereas *δ* defines the interval between two series of the study.

The *AcquisitionTime* attribute indicates the time the scanner starts the acquisition of data that resulted in the image. Although the AcquisitionTime attribute is defined universally across modalities, the *SeriesTime* attribute is not standardized. For example, the SeriesTime attribute indicates the time the series ended for MR scanners, but it indicates the series start time for the CT scanners.

We formulate the series attributes as follows. $\forall \chi \;\in {\mathbb{Z}}^{+}\;:\;t_{s}x$ represents the acquisition time of the χ^*th*^ image in each series *s*_*i*_, belonging to a study *S*_*i*_, and |*s*_*i*_| represents the number of images in the series *s*_*i*_. *Niffler* computes the series duration *υ*_*i*_ as the time duration between the AcquisitionTime of the first and the last images of the series, as shown by Equation 2. There are vendor-specific private tags that provide the series end time, which can replace $t_{s_{i}^{\left|s_{i}\right|}}$ to compute a more accurate *υ*_*i*_.

(2) $$v_{i}=\left[t_{s_{i}^{1}},t_{s_{i}^{\left|s_{i}\right|}}\right]$$

*S*_*ijk*_ represents *i*^*th*^ study for the patient *p*_*j*_ from the scanner *m*_*k*_. The study start time (*τ*_0_) and the study end time (*τ*_*f*_) are calculated as shown by Equation 3. *Niffler* considers study start time as the acquisition time of the first image of the first series, and the study end time as the acquisition time of the last image of the last series.

(3) $$\forall i\in {\mathbb{Z}}^{+},\forall s_{i}\in S_{i}\;:\;\tau_{0}^{S_{i}}=\text{min}\;t_{s_{i}^{1}},\;\tau_{f}^{S_{i}}=\text{max}\;t_{s_{i}^{\left|S_{i}\right|}}$$

For performance reasons, *Niffler*, by default, considers only the first image in each series when extracting the metadata, approximating the study end time as Equation 4 shows. Hence, the default deployment approximately calculates the study end time as the acquisition time of the first image of the last series.

(4) $$\forall i\in {\mathbb{Z}}^{+},\forall s_{i}\in S_{i}\;:\;\tau_{0}^{S_{i}}=\text{min}\;t_{s_{i}^{1}},\;\tau_{f}^{S_{i}}\approx \text{max}\;t_{s_{i}^{1}}$$

We define *d*(*S*_*i*_, *p*_*j*_, *m*_*k*_) as the duration of study *S*_*i*_ of patient *p*_*j*_ in scanner *m*_*k*_. In a timescale, the study duration is the time between the study start time and the study end time as Equation 5 shows.

(5) $$\forall i,j,k\in {\mathbb{Z}}^{+},d\left(S_{i},p_{j},m_{k}\right)=d_{ijk}=\left[\tau_{0}^{S_{ijk}},\tau_{f}^{S_{ijk}}\right]$$

A scanner produces several series for each study *S*_*i*_, during the time duration of ($\tau_{f}^{S_{ijk}}-\tau_{0}^{S_{ijk}}$). Considering the *υ*_*i*_ for each series in the study, Equation 6 shows the sum of series interval *δ* between the series in a study *S*_*i*_.

(6) $$\sum_{S_{i}}\delta =\left(\tau_{f}^{S_{i}}-\tau_{0}^{S_{i}}\right)-\sum_{\forall s_{i}\in S_{i}}v_{i}$$

For the studies to have the potential to be part of the same encounter, there should not be another patient seen by the scanner in between two consecutive studies for the same patient. Equation 7 defines this constraint. The duration *d*_*lnk*_ is of the study *S*_*l*_ of patient *p*_*n*_ seen by the scanner *m*_*k*_. *m*_*k*_ is the same scanner that sees the patient *p*_*j*_. *d*_*lnk*_ does not overlap with the time interval between the earliest scan time of ψ_*ijk*_ and ψ_*rjk*_ and the latest scan time of ψ_*ijk*_ and ψ_*rjk*_. Here, we identify the studies and encounters with auto-incrementing numbers. In the following equations, we assume that this constraint is satisfied.

(7) $$\forall l,n\in {\mathbb{Z}}^{+},p_{j}\neq p_{n},r=i\pm 1\;∄\left[\text{min}\left\{\tau_{0}^{\Psi_{ijk}},\tau_{0}^{\Psi_{rjk}}\right\},\;\text{max}\left\{\tau_{f}^{\Psi_{ijk}},\tau_{f}^{\Psi_{rjk}}\right\}\right]\cap d_{\text{lnk}}\neq \emptyset$$

We define a constant, *tolerance interval* (*I*), as a specified maximum time interval between any two consecutive studies of the same patient from the same scanner for them to be considered as belonging to the same encounter. In other words, *Niffler* considers multiple studies of the same patient on the same scanner with no other patient’s study in between (Equation 7) a single encounter if they have a time interval less than *I*. *I* is 20 minutes in our deployment and also the default value of *Niffler*.

*Niffler* identifies those encounters seamlessly on-the-fly, by merging such studies as Equation 8 illustrates, subject to the constraints presented by Equations 7, 9. Here *Niffler* retrospectively looks at the studies from the past few minutes to find overlapping studies as they indeed belong to a single encounter.

(8) $$d\left(E_{i},\;p_{j},\;m_{k}\right)=\left[\text{min}\left\{\tau_{0}^{\Psi_{ijk}},\;\tau_{0}^{\Psi_{rjk}}\right\},\;\text{max}\left\{\tau_{f}^{\Psi_{ijk}},\;\tau_{f}^{\Psi_{rjk}}\right\}\right]$$

(9) $$s.t.:\;\left[\tau_{0}^{\Psi_{ijk}}-I/2,\;\tau_{f}^{\Psi_{ijk}}+I/2\right]\cap \;\left[\tau_{0}^{\Psi_{rjk}}-I/2,\;\tau_{f}^{\Psi_{rjk}}+I/2\right]\neq \emptyset$$

When two studies of the same patient from the same scanner have a time interval of *I* or more, we consider such two studies to be two different encounters, as shown by Equation 10, subject to the constraints presented by Equations 7, 11.

(10) $$d\left(E_{i},\;p_{j},\;m_{k}\right)=d_{ijk};\;d\left(E_{r},\;p_{j},\;m_{k}\right)=d_{rjk}$$

(11) $$s.t.:\;\left[\tau_{0}^{\Psi_{ijk}}-I/2,\;\tau_{f}^{\Psi_{ijk}}+I/2\right]\cap \;\left[\tau_{0}^{\Psi_{rjk}}-I/2,\;\tau_{f}^{\Psi_{rjk}}+I/2\right]=\emptyset$$

Equation 12 presents the computation of scanner utilization *U*_*k*_ for scanner *m*_*k*_, at the encounter level. $T_{0}^{E_{i}}$ and $T_{f}^{E_{i}}$ indicate the start and end times of an encounter *E*_*i*_. Γ_*k*_ represents the time the scanner *m*_*k*_ remains switched on for the considered duration. We approximate Γ_*k*_ to be the time between the first and last scans by the scanner during the considered duration. As *Niffler* considers a day as the default duration, Γ_*k*_ becomes the time between the first and last scan of the day by the scanner.

(12) $$\forall E_{ijk}\in m_{k}:U_{k}=\frac{\sum d_{ijk}}{\Gamma_{k}}\approx \frac{\sum d_{ijk}}{\text{max}\;T_{f}^{E_{ijk}}-\text{min}\;T_{0}^{E_{ijk}}}$$

The vision for optimizing scanner utilization is to minimize both the study intervals and the series intervals. Equation 13 presents our motivation to minimize the intervals Δ between each encounters, to increase the scanner utilization as defined by Equation 12.

(13) $$\text{minimize }\sum_{\Gamma_{k}}\left(\tau_{0}^{E_{i+1,j,k}}-\tau_{f}^{E_{i,j,k}}\right)\;\forall E_{i,j,k},E_{i+1,j,k}\in m_{k}.$$

Finally, Equation 14 presents our motivation to reduce the series intervals *δ* between series belonging to the same encounter, extending the Equation 6.

(14) $$\text{minimize }\sum_{\Gamma_{k}}\left(\left(\tau_{f}^{S_{i}}-\tau_{0}^{S_{i}}\right)-\sum_{\forall s_{i}\in S_{i}}v_{i}\right)$$

In addition to improving scanner utilization, Δ also aims to enhance the patient experience through minimal patient wait times by reducing the study intervals. Reducing *δ* ensures shorter examination times and a high scanner utilization from the system’s perspective. Series intervals are much smaller in scale, thus making it harder to reduce them further. Hence, we focus on reducing the study intervals in this work.

### C. ALGORITHMS

*Niffler* consists of two algorithms that perform the scanner utilization computations. First, Algorithm 1 aggregates the scanner properties for the specified period. For simplicity, we discuss the default case that assumes that computation is for the studies completed the day before. The algorithm produces metaMap, a map consisting of the number of series in the study, study start time, study end time, and study duration, in addition to the selected attributes from the DICOM metadata. Algorithm 2 illustrates the scanner utilization algorithm, which computes and produces the scannerMap as the second step. As a prerequisite, it invokes the Algorithm 1 and retrieves the *metaMap* for the previous day (or the specified time interval). The output of the Algorithm 2 is a *scannerMap*, which lists the scanners, whose each entry further consists of an *encounterMap* that presents the properties of each encounter belonging to the scanner.


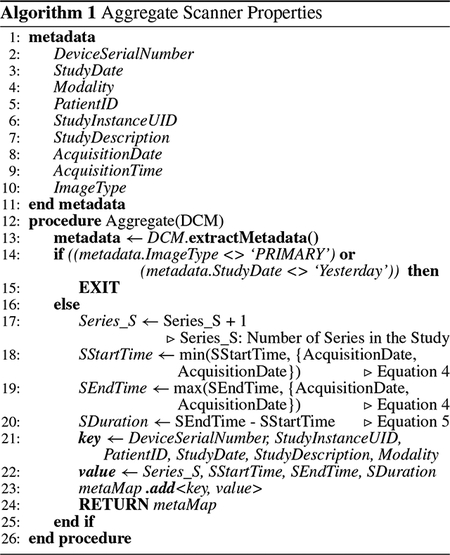


#### 1) SCANNER PROPERTIES AGGREGATION

Algorithm 1 first gets the relevant metadata attributes from the DICOM image (line 13). It then filters for the ImageType and the specified duration (line 14). Then, for each matching entry, the series count is increased by one, as *Niffler* by default extracts only the first image of a series (line 17). However, when we configure *Niffler* to extract metadata from all the images, the count will indicate the image count. In that case, *Niffler* must compute the series count as the number of entries with unique SeriesInstanceUIDs. Following the Equations 4 and 5, the algorithm calculates the study start time (SStartTime), study end time (SEndTime), and study duration (SDuration) (lines 18 – 20).

The DICOM properties DeviceSerialNumber, StudyInstanceUID, PatientID, StudyDate, StudyDescription, and Modality compose the key of the metaMap (line 21). The calculated properties Series_S, SStartTime, SEndTime, and SDuration compose the value of the metaMap (line 22). The algorithm (line 23) adds each key-value pair to the metaMap as an entry. Finally, it returns metaMap as its output (line 24) to be processed further by Algorithm 2 to produce the scanner utilization metrics.

#### 2) SCANNER UTILIZATION COMPUTATION

Algorithm 2 loops through the metaMap to compute the final properties that compose the scannerMap (line 6). StudyDate and DeviceSerialNumber compose the scannerMap’s key (*scannerKey*) (line 7). The algorithm defines a temporary variable *eStatus* to identify whether the study belongs to an existing patient and should it be merged into an existing encounter or created as a separate encounter for the same patient (lines 11 – 13). The attribute EncounterID uniquely identifies the encounter based on the eStatus (lines 14 – 15). *Niffler* defines EStartTime, EEndTime, and EDescription from SStartTime, SEndTime, and StudyDescription, respectively, based on the eStatus, to represent the start time, end time, and the description of the encounter (lines 16 – 19). It defines the encounter duration (EDuration) as the difference between EEndTime and EStartTime (line 20). *Patients_S_D, Encounters_S_D*, and *Series_S_D* indicate the counts for patients, encounters, and series respectively for the scanner for the defined duration (lines 21 – 24). *Studies_E* and *Series_E* depict the number of studies and series in the encounter (lines 25 – 27).

The encounterMap uses the encounterID as its key. The properties – EStartTime, EEndTime, EDuration, Studies_E, Series_E, and EDescription, compose the encounter-Value, the values for the encounterMap (line 28 – 29). The algorithm adds the key-value pair to the encounterMap (line 30). It then computes the scanner utilization (ScannerUtil) from the encounterMap (line 31). It then adds the scanner-specific aggregate properties to the scannerMap’s value (*scannerValue*): Modality is included as is, together with other computed properties such as ScannerUtil, Patients_S_D, Encounters_S_D, Studies_S_D, Series_S_D, and the encounterMap (lines 32 – 34). Finally, it adds the scannerKey-scannerValue pair to the scannerMap and returns scannerMap as the output (lines 35 – 36).

The *Niffler* architecture enables efficient processing of metadata for various workflows, including understanding scanner utilization. The formulated equations and their implementation as the scanner utilization computation algorithm facilitate understanding of key performance metrics.

## IV. EVALUATION

We deployed *Niffler* on a server of 16 GB memory, Centos 7 operating system, 32 TB disk space, and AMD Opteron 63xx class * 4 CPUs. The *Niffler* deployment has been running stable for over the past two years, receiving images in real-time from our extensive healthcare network’s enterprise PACS with minimal data loss. The data included images belonging to various modalities, up to 350 GB/day from 715 scanners that span 12 sites. We benchmarked the effectiveness of *Niffler* in identifying scanner utilization metrics against RIS, using our 25 MRI scanners, during four weeks starting from June 1st, 2020 (i.e., from Monday, June 1st - Sunday, June 28th). We observe and understand the scanner utilization patterns daily over the period, individually for each scanner and cumulatively for all the 25 scanners.


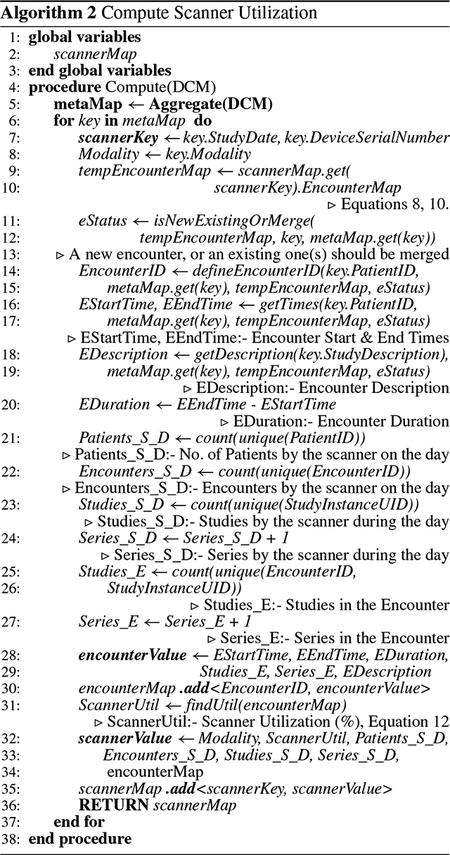


### A. UTILIZATION AND ENCOUNTERS OF EACH SCANNER

**FIGURE 6.**
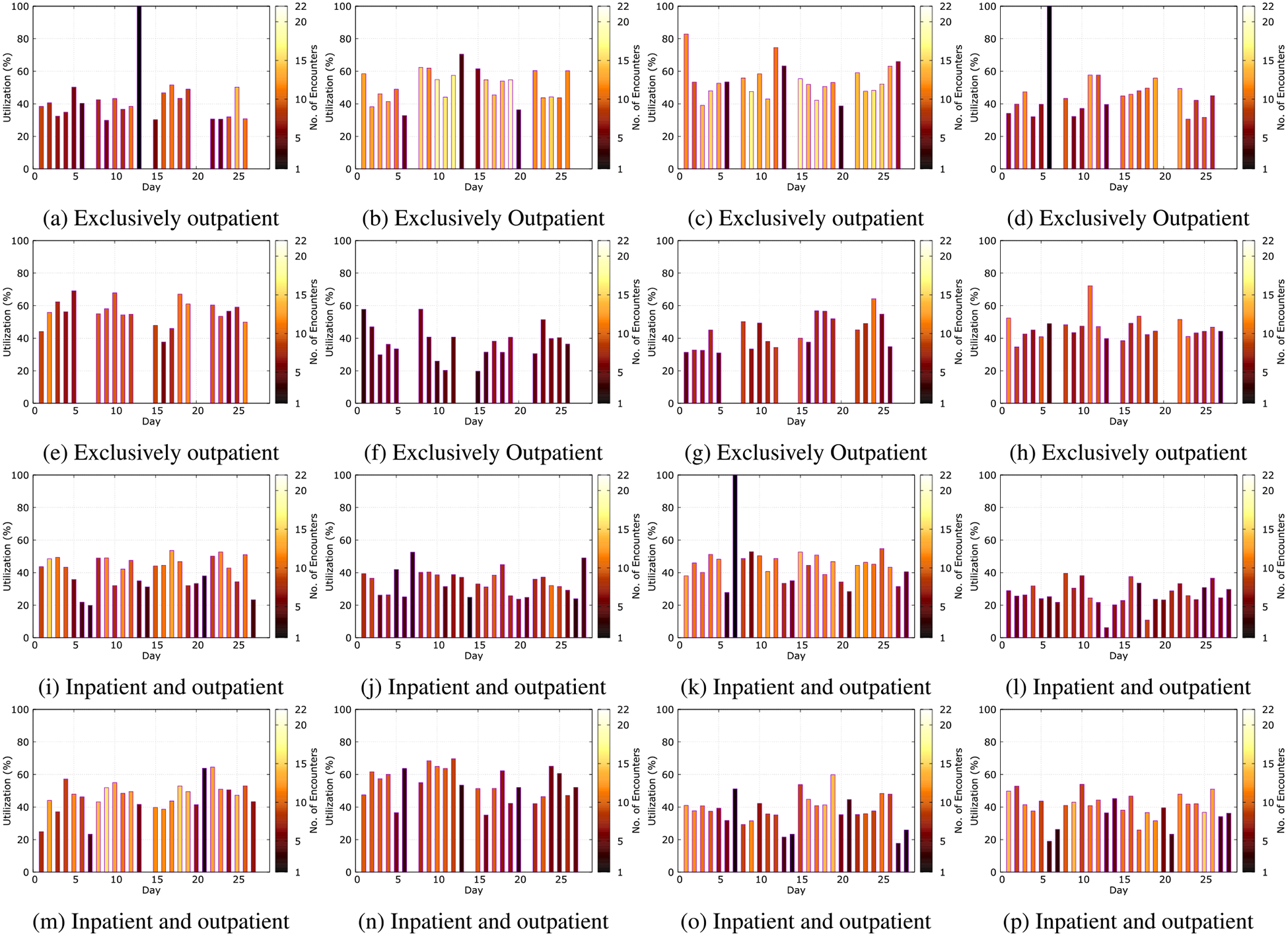
Scanner utilization and the number of encounters, as reported by *Niffler* for each scanner.

First, we observe the utilization and the number of encounters for each scanner as reported by *Niffler*. These parameters are not readily available from RIS. Figure 6 illustrates these parameters of selected 16 scanners among the 25 MRI scanners. Figures 6a, 6b, 6c, 6d, 6e, 6f, 6g, and 6h represent the scanners of exclusively outpatient usage, whereas the other 8 represent the scanners that scan both inpatients and outpatients. The bars indicate the utilization parameter that we compute for each scanner as a percentage, as illustrated by Equation 12. The colors of the bar indicate the number of encounters. The lighter shades indicate more encounters, whereas the darker shades indicate fewer encounters.

The utilization parameter gives a more accurate representation when the scanner performs more encounters, spread across the day. Fewer encounters lead to a less useful value for the utilization properties. Mainly if the scanner performs only one encounter for the day, the utilization is reported as 100%. Overall, from Figure 6, we notice the typical patterns of scanner usage over the days of the week. The scanners performed fewer encounters on Saturdays and Sundays. Some scanners, such as the ones depicted by Figure 6e, 6f, and 6g, did not perform any exams at all during the weekends. The remaining outpatient scanners performed fewer scans on Saturdays and did not perform any encounters on Sundays.

The plots confirm our healthcare network’s characteristics that the inpatient-outpatient scanners have more distributed appointments spread across all the days. On the other hand, exclusively outpatient scanners have a more structured schedule. The usage pattern, including the calculated scanner utilization parameter and the number of encounters, are well-spread across the days. Our observations highlight how *Niffler* can be used to demonstrate and understand the scanner utilization parameters more effectively.

### B. STUDIES AND ENCOUNTERS ACCUMULATED ACROSS ALL THE SCANNERS

**FIGURE 7.**
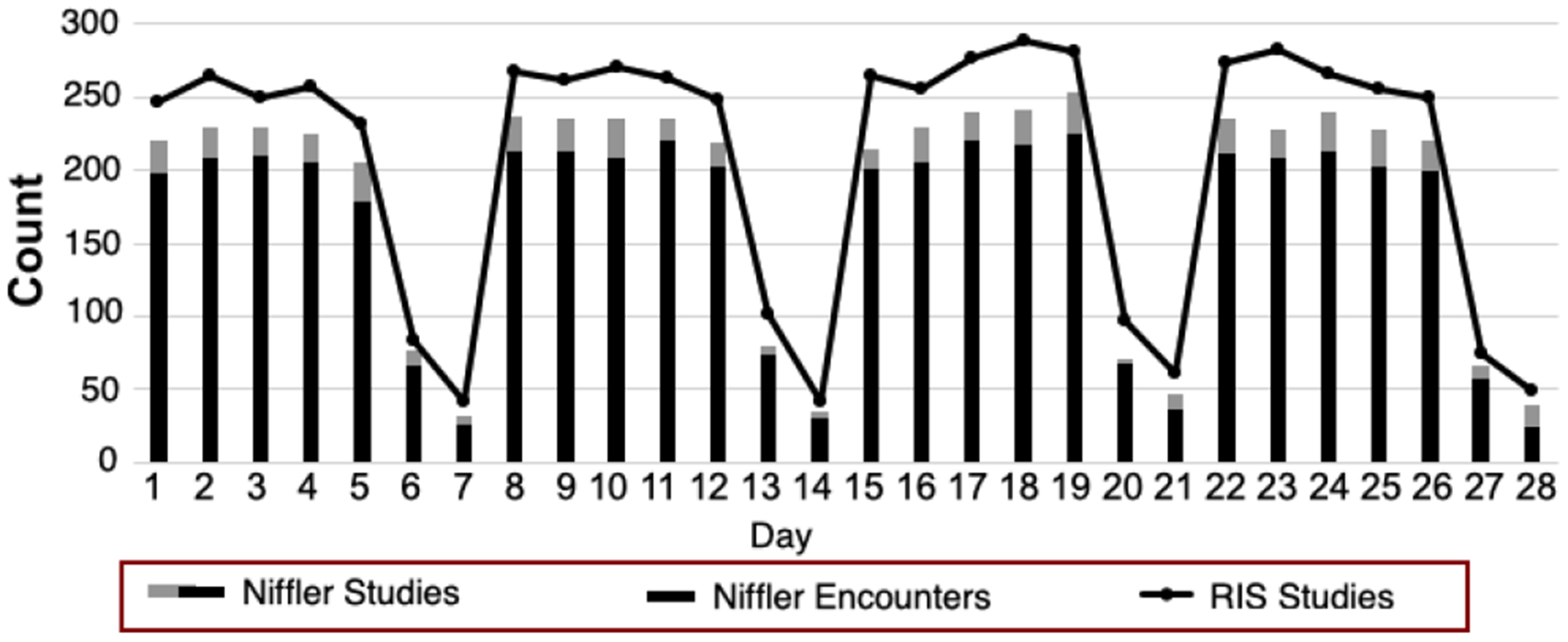
Number of Studies observed from *Niffler* and RIS.

We then compare the number of studies and encounters observed by RIS and *Niffler* for each day, accumulated for all the 25 MRI scanners. Figure 7 reports the number of studies from *Niffler* and RIS, as well as the number of encounters from *Niffler*. The bars indicate the observations by *Niffler*: the black bars indicate the number of encounters, whereas the combinations of both the black and gray bars show the number of studies. The line chart shows the number of studies reported by RIS. While *Niffler* accurately identifies and merges multi-exam encounters, RIS does not possess that capability. As such, *Niffler* outperforms RIS with its accurate representation of encounters.

However, overall, RIS provides a more accurate count of studies compared to *Niffler*. *Niffler* reported fewer studies for all the days, due to studies with no images in them and images with missing mandatory DICOM headers. First, *Niffler* performs analyses based on the DICOM headers of the received images. Occasionally, scanners produce valid studies and series with no images associated with them, holding only textual series-specific metadata, stored as file types other than DICOM images. But since *Niffler* entirely relies on DICOM headers for its computations, *Niffler* Metadata Store does not store details of such studies. Second, *Niffler* scanner utilization computation algorithms ignore any image that misses certain specific mandatory DICOM attributes necessary for the computations. Due to these two reasons, RIS reported more studies than *Niffler* and provided a more accurate count of studies.

As studies with zero images are typically followed by or preceded by a study with DICOM images for many multi-exam encounters, we note that *Niffler* provides a more accurate count of encounters even though it reported fewer studies. Specific private DICOM attributes may help us identify encounters with multiple studies, which present zero images on PACS. For example, images scanned by the MRI scanners of Siemens consist of a private tag called ‘Body Part,’ often tied to the protocol selected by the radiographer. However, such private tags are specific to the vendor and are not represented across multiple vendors uniformly. Therefore, using private DICOM attributes to identify scanner performance parameters is not a scalable approach.

### C. SCAN DURATION

**FIGURE 8.**
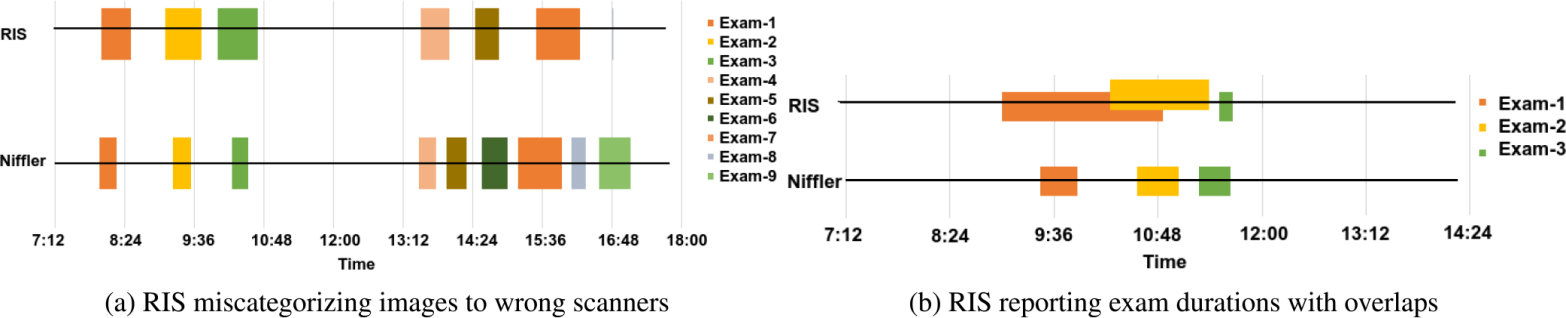
Exam durations by 2 scanners on a day as reported by *Niffler* and RIS.

We finally compare the exam durations as reported by *Niffler* and RIS. Figure 8 illustrates the scan durations for two scanners for their operation during the observed day. We note that *Niffler* provided a more accurate representation of the duration as the actual time is recorded in the metadata. On the other hand, RIS depicted an inaccurate timeline. Figure 8a shows that RIS has failed to correctly associate two out of the nine exams to the scanner. An exam (Exam-8) is also wrongly timed by RIS at 16:48 as a very brief exam. Figure 8b shows that RIS reported overlapping exams by the same scanner, which is not possible in practice as one scanner can carry out only one exam at any given time. *Niffler* correctly identified the scan durations, with no overlaps and no miscategorization of exams to the scanners.

Overall, we observed that *Niffler* illustrates an accurate representation of the encounter durations in any given scanner across all the days, whereas RIS encountered several human errors. This observation is further highlighted as RIS reported overlapping encounters and misattributed a few studies to wrong scanners. Furthermore, RIS does not possess the ability to correctly identify the multi-exam encounters, thus introducing further inaccuracies in its representation.

#### 1) SCANNER MISREPRESENTATIONS IN RIS

When a patient is rescheduled and seen by another scanner than the one they are initially assigned to, the information is often not updated in the RIS. Human errors cannot also be neglected when two scanners have near-identical names. For example, we noticed for a day, RIS reported the scanner *MR RM 1 CL* to have visibly too many encounters, wrongly attributing some encounters from *MR RM 2 CL* to *MR RM 1 CL*. Among the 28 days that we observed, we note from 10 – 13% of misrepresentation of scanners for exams by RIS. This human error wrongly categorized the scanner that performed a scan. For example, 33 out of 244 exams were misrepresented in the RIS across the 25 MRI scanners in a day. Most of these were due to the rescheduling of examinations across the scanners that were not updated in the RIS. On the other hand, the scanner’s information, as recorded by the DICOM metadata, is always accurate. As such, we note that human errors dominate the calculations made based on the data from RIS, whereas machine errors (errors beyond direct human actions) dominate the *Niffler* calculations. We note that *Niffler* and PACS-based approaches provide accurate representations of scanner utilization, as they avoid the human errors of miscategorization.

#### 2) ACCURACY IN STUDY DURATIONS

In the metadata extractions and the subsequent evaluations, our *Niffler* deployment considers only the first images of each series. We chose to extract the metadata only from each series’s first image entirely due to performance considerations. For deployments with sufficient computational resources to handle the higher loads with spikes, we can quickly reconfigure the *Niffler* Metadata Extractor to extract the metadata from all the images of each series. In that case, *Niffler* could more accurately represent the study end time by the acquisition time of the last image of the last series belonging to the study.

#### 3) DATA LOSS

Data loss can be a challenge in *Niffler* deployments. Data loss may typically happen when the DICOM images are sent from the scanners to the PACS or from the PACS to the downstream DICOM listeners such as *Niffler*. Previous works have reported data loss between the scanners and PACS due to various reasons such as PACS network overload and vendor incompatibility that arises when the scanners or the PACS use unsupported vendor-specific options beyond the DICOM standard reference implementation [9]. During brief downtimes, the PACS in our radiology department maintains a queue and retries to send the series to the downstream DICOM listeners such as *Niffler*. As such, data loss between the PACS and *Niffler* is minimized. However, data loss occurs when the queue builds up for several days due to prolonged outages (such as hardware, network, or software failure) in the receiving end downstream. Furthermore, more data loss will occur during data transfer failures if the PACS is not configured to maintain a queue of failed series to resend them. We did not observe any data loss with our stable deployment during our observed timeframe of 28 days in June.

#### 4) TRUE STUDY TIMES

A challenge with using PACS to find the encounter duration is, the actual start time and end time of an examination are never known. The time *Niffler* identifies is based on what is sent to the PACS as DICOM images and does not account for the true (early) start or true (late) end of an encounter beyond the DICOM image acquisitions received by PACS. On the other hand, data acquired from RIS may provide a more accurate representation of the true start and end times of an encounter, as long as no human error is introduced in recording the times. However, as human mistakes are more common, in practice *Niffler* still produces a better representation of the study duration. Furthermore, *Niffler* algorithms correctly find the durations of multi-exam encounters, an impossible endeavor with RIS or PACS alone.

## V. DISCUSSION

This section discusses the design choices of *Niffler*, their benefits, and the shortcomings in its PACS-based approach.

The tolerance interval plays a significant role in defining an encounter by merging multiple consecutive studies. When we increase the tolerance interval (for example, 40 minutes instead of the considered 20 minutes), the number of encounters becomes equal to the number of patients for more cases, as more studies are identified as the same encounter. On the other hand, when we decrease it (for example, 15 minutes instead of 20 minutes), the number of encounters becomes closer to the number of studies, as less of the studies are merged into an encounter. We chose the 20 minutes default value by analyzing the study durations and the typical intervals between two studies that should be considered the same encounter in our healthcare network. We can manually change this value based on the healthcare networks: precisely, the observed study intervals between the studies of the same encounter of the scanners. Using the scanner utilization patterns as observed by *Niffler* to adjust the tolerance interval without manual intervention dynamically is future work.

PACS sometimes receives a few images from the scanners later than the rest, breaking the assumption of real-time transfers. We avoid such delays by computing the scanner utilization at 02:00 a.m. for the previous day. If we perform the scanner utilization at a pseudo real-time, such delays will affect the computations for computations based on both RIS and PACS due to the delays in receiving images. We can currently trigger to compute the retrospective data from *Niffler* to account for later changes in the past data daily. Similarly, a more frequent execution can be scheduled to recompute the scanner utilization with newly arrived data so that the computations will provide a more accurate vision of the scanner utilization trend. As future work, we aim to extend *Niffler* to track newly arrived DICOM series from the past and trigger the scanner utilization computations to update the previous calculations more dynamically.

Several circumstances lead to a higher or lower number of reported studies in the PACS than their true count. For example, an appointment may lead to 2 or (rarely) more studies due to patient error, such as patient movements that prevented the image acquisition seamlessly. Although they might be represented as multiple studies in PACS, *Niffler* will merge those when identifying the encounters. However, studies with zero images of ImageType ORIGINAL are not accounted for in the number of studies or encounters by *Niffler*. Sometimes two studies are reported as one study with a large number of series and the other with 0 series. In this case, *Niffler* ignores the study with 0 series. While this affects the number of studies, we note that such studies with 0 series do not affect scanner utilization. However, the study description belonging to the study with 0 series is lost in that case. We also note that scanner utilization is not a standardized reference unit and is up to different interpretations.

## VI. CONCLUSION

Accurately measuring scanner utilization metrics can minimize patient wait times and examination times while optimizing the scanners’ performance. However, the radiology departments lack resources to compute scanner utilization metrics efficiently. Typically, radiology departments use data from RIS to calculate scanner usage statistics. However, data derived from RIS are inaccurate due to human errors and inadequate as RIS data is not as rich as the DICOM metadata. On the other hand, extracting DICOM metadata to compute various utilization metrics would require computation capabilities lacking in the radiology departments.

In this paper, we presented *Niffler*, a framework with a PACS-based approach and algorithms to compute scanner utilization metrics more accurately. The proposed *Niffler* framework transfers DICOM images from the PACS to a research cluster in real-time and extracts and processes the DICOM metadata in a pseudo real-time to understand scanner utilization metrics. Evaluations of *Niffler* on our extensive healthcare network highlight how metrics obtained and processed from the metadata headers can be more accurate in estimating scanner utilization than the data obtained from the RIS that rely on the manually entered data.

As future work, we aim to compute the tolerance interval dynamically for each scanner, thus facilitating a more accurate definition of encounters across multiple healthcare networks and modalities. Finally, we propose incorporating external operational measures such as refining the scheduled exam timeslots and improving protocol standardization across scanners and institutions based on system performance and utilization. Thus, we aim to minimize patient exam times and wait times across healthcare networks. We posit *Niffler* PACS-based approach as complementary to the real-time data readily available from RIS, rather than to replace it. As such, we note that, combined with the clinical data from the RIS, *Niffler* can produce a more accurate and complete analysis compared to using RIS or PACS alone.

## Footnotes

1 The source code of *Niffler* can be found at https://github.com/Emory-HITI/Niffler
